# Retention Enhancement in Low Power NOR Flash Array with High-κ–Based Charge-Trapping Memory by Utilizing High Permittivity and High Bandgap of Aluminum Oxide

**DOI:** 10.3390/mi12030328

**Published:** 2021-03-19

**Authors:** Young Suh Song, Byung-Gook Park

**Affiliations:** 1 Department of Electrical and Computer Engineering, Seoul National University, Seoul 08826, Korea; sys1413@snu.ac.kr; 2 Department of Computer Science, Korea Military Academy, Seoul 01805, Korea

**Keywords:** retention characteristic, high-κ, nonvolatile charge-trapping memory, stack engineering, NOR flash memory, aluminum oxide

## Abstract

For improving retention characteristics in the NOR flash array, aluminum oxide (Al_2_O_3_, alumina) is utilized and incorporated as a tunneling layer. The proposed tunneling layers consist of SiO_2_/Al_2_O_3_/SiO_2_, which take advantage of higher permittivity and higher bandgap of Al_2_O_3_ compared to SiO_2_ and silicon nitride (Si_3_N_4_). By adopting the proposed tunneling layers in the NOR flash array, the threshold voltage window after 10 years from programming and erasing (P/E) was improved from 0.57 V to 4.57 V. In order to validate our proposed device structure, it is compared to another stacked-engineered structure with SiO_2_/Si_3_N_4_/SiO_2_ tunneling layers through technology computer-aided design (TCAD) simulation. In addition, to verify that our proposed structure is suitable for NOR flash array, disturbance issues are also carefully investigated. As a result, it has been demonstrated that the proposed structure can be successfully applied in NOR flash memory with significant retention improvement. Consequently, the possibility of utilizing HfO_2_ as a charge-trapping layer in NOR flash application is opened.

## 1. Introduction

With the advent of the Fifth Generation Mobile Networks (5G) era, the demand for big data has increased rapidly in recent years [1,2,3], and the need for memory devices enabling more data storage has consistently increased [4,5]. In order to satisfy these demands, novel memory devices utilizing new materials such as aluminum oxide (Al_2_O_3_, alumina), hafnium oxide (HfO_2_), zirconium dioxide (ZrO_2_), stacked HfO_2_/Al_2_O_3_, and nano-laminated forms (HfAlO_x_) have been widely proposed and studied [6,7,8].

Among them, hafnium oxide (HfO_2_) has a tremendous advantage as a charge-trapping layer (CTL) material, since its charge trap density is four times higher than that of the conventional charge-trapping layer (CTL), silicon nitride (Si_3_N_4_) [9,10]. This enriched CTL density of HfO_2_ can enable a wider threshold voltage (*V*_TH_) window and improved memory margin [11,12]. Furthermore, permittivity of HfO_2_ is much higher than that of Si_3_N_4_, which enables significant reduction in equivalent oxide thickness (EOT) of the gate stack [13,14,15,16,17]. This enables low program voltage (*V*_PGM_), low erase voltage (*V*_ERS_), fast program/erase (P/E) speed, fast switching speed, and low power consumption. 

From these various advantages of higher charge trap density and the possibility of reducing EOT, HfO_2_-based charge-trapping memories (CTM) have been widely studied for fast, high-capacity nonvolatile memory devices [18,19,20,21]. However, despite these advantages, HfO_2_ has encountered many limitations in commercialization due to retention problems that come from its numerous shallow traps [22,23,24,25]. Therefore, this issue needs to be solved for realizing practical high-κ–based charge-trapping memory (HCTM).

In order to solve these retention issues, the use of Al_2_O_3_ as a CTL in a metal/Al_2_O_3_/SiO_2_/Si (MAOS) structure has been proposed, but it also suffers from retention problems due to vertical leakage current [26,27]. Another previous solution of simply increasing the thickness of tunneling oxide layers has been proposed to mitigate this retention problem; however, this approach concomitantly results in the degradation in P/E speed and subthreshold swing (SS) due to an increase in EOT of the gate stack [28,29,30,31]. Furthermore, this approach inevitably increases *V*_PGM_, *V*_ERS_, and power consumption. Therefore, a new approach is needed to solve these issues.

In this framework, the aim of this paper is to 1) improve retention characteristics of HfO_2_-based CTM by using tunneling oxide layers of SiO_2_/Al_2_O_3_/SiO_2_ and 2) validate that our proposed structure can be well applied in the NOR flash array, which has been broadly studied for unsupervised learning [32,33]. For validating retention improvement in the proposed memory device structure, it is also compared with the other bandgap engineering (BE) tunneling oxide layers with SiO_2_/Si_3_N_4_/SiO_2_ [34,35,36].

Consequently, it has been demonstrated that the retention characteristics can be significantly improved in a high-κ–based NOR flash memory device by utilizing the advanced tunneling layers with SiO_2_/Al_2_O_3_/SiO_2_ on the tunnel field effect transistor (TFET) structure, which has been broadly studied for low power application [37,38,39,40,41,42,43,44]. From an array perspective, it has been demonstrated that the proposed memory device structure is also able to inhibit the programming in unselected cells by bottom gate effect. Namely, we have designed the memory device structure which is free from disturbance issues in the NOR flash array with enhanced retention characteristics.

This paper is organized as follows. First, the basic transfer characteristics are analyzed after calibration. Second, performance of inhibition in the NOR flash array is demonstrated. Then, improvement of the retention characteristics is carefully analyzed with various perspectives. Finally, the expected advantage of applying our proposed memory device structure in the NOR flash array is discussed.

## 2. Device Structure and Model Physics

### 2.1. Structure of the Proposed Memory Device

In previous research, the advanced bandgap-engineered TaN/Al_2_O_3_/HfO_2_/SiO_2_/Si (BE-TAHOS) structure has been investigated for a faster erasing speed and larger memory window by incorporating Si_3_N_4_ at the tunneling oxide layer [37,38,39,40,41,42,43,44]. By utilizing this BE-TAHOS structure [34,35,36] and applying Al_2_O_3_ at the tunneling layer, the advanced structure of TaN/Al_2_O_3_/HfO_2_/SiO_2_/Al_2_O_3_/SiO_2_/Si (TAHOAOS) is designed for NOR flash memory.

**Figure 1:**
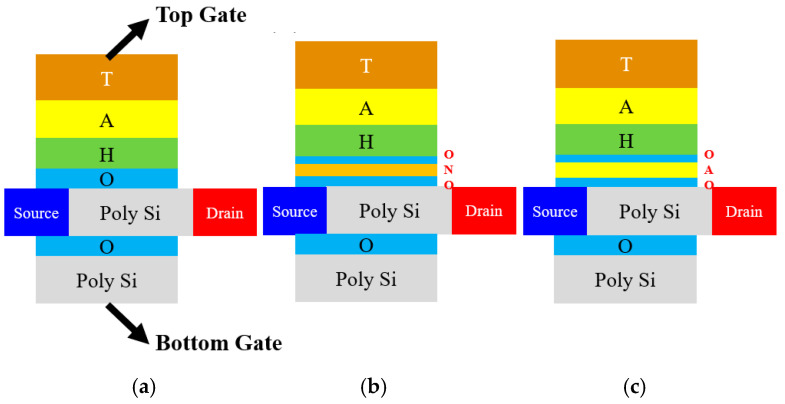
Schematic view illustrating (**a**) conventional TaN/Al_2_O_3_/HfO_2_/SiO_2_/Si (TAHOS), (**b**) bandgap engineered (BE)-TAHOS, and (**c**) proposed TaN/Al_2_O_3_/HfO_2_/SiO_2_/Al_2_O_3_/SiO_2_/Si (TAHOAOS) structure with two gate terminals. All structures commonly have HfO_2_ as charge-trapping layer (CTL) and Al_2_O_3_ as blocking oxide. The abbreviated letters T, A, H, O, N stand for tantalum nitride (TaN, gate metal), Al_2_O_3_, HfO_2_, SiO_2_, Si_3_N_4_, respectively.

Cross-sectional views of conventional TaN/Al_2_O_3_/HfO_2_/SiO_2_/Si (TAHOS), BE-TAHOS, and that of the proposed TAHOAOS structure are schematically shown in Figure 1. In order to compare the proposed TAHOAOS structure with not only conventional TAHOS but also the BE-TAHOS structure, BE-TAHOS is also designed with SiO_2_/Si_3_N_4_/SiO_2_ tunneling oxide layers [34,35,36]. The devices designed in this work have four terminals with top gate, bottom gate, source, and drain. The bottom gate is designed for solving disturbance issues.

Table 1 describes the film thickness and channel length for these devices. The simulated devices are composed of tunneling oxide layers with the same EOT of 3 nm for fair comparison. The blocking oxide is composed of 6 nm Al_2_O_3_, and CTL is composed of 4 nm HfO_2_. Bottom gate dielectric has a 3 nm thickness with SiO_2_. The length and thickness of the silicon channel are 40 nm and 12 nm, respectively. A gate-drain underlap (gate-source overlap) structure is applied for suppressing ambipolar current [38,39], which undesirably increases the off-state current. In specific, since the ambipolar current occurs due to band-to-band-tunneling (BTBT) current in the body/drain region, it is possible to suppress the ambipolar current by locating the gate far from the drain, which is called gate-drain underlap [38,39].

### 2.2. Model Physics and Model Parameters

To carefully investigate the electrical characteristics in these three different structures, tunneling models such as band-to-band-tunneling (BTBT), Fowler-Nordheim (FN) tunneling, direct tunneling, and trap-assisted tunneling (TAT) are applied in this device simulation with Synopsys Sentaurus™ through a technology computer-aided design (TCAD) tool. Physical models including Shockley-Read-Hall (SRH) recombination and E-field saturation models are also applied for precisely analyzing the memory operation.

For details, we adopted various mobility models including the PhuMob mobility model, Enormal (Lombardi) mobility model, and thin-layer mobility model to consider interfacial surface calibration roughness scattering and Coulomb scattering. In addition, models of eHighFieldSaturation, hHighFieldSaturation, and Avalanche (CarrierTempDrive) are used for reflecting velocity saturation and avalanche breakdown. Non-local mesh, eBarrierTunneling, and hBarrierTunneling are utilized for applying FN tunneling and direct tunneling.

In modeling HfO_2_ as CTL, charge trap density of 1.2 × 10^20^ cm^−^^3^ is applied for HfO_2_, which corresponds to its charge trap density in memory device [9,10,11]. Specifically, the energy depth of electron is set as 0.7 eV from the lowest conduction band (LCB) of HfO_2_ [20], whereas the energy depth of hole is set as 2.9 eV from the highest valence band (HVB) [21] of HfO_2_. On the other hand, in modeling Al_2_O_3_, charge trap density of 2.0 × 10^12^ cm^−^^3^ is applied, and the energy depth of electron/hole is set as 0.4/2.7 eV from LCB/HVB, respectively [8]. In addition, effective electron tunneling masses (*m*_eff_) of 0.55 m_o_, 0.2 m_o_, and 0.4 m_o_ are used in thin film of SiO_2_ [12], HfO_2_ [12], and Al_2_O_3_ [17], respectively.

### 2.3. Workflow of Study and Calibration Process

**Figure 2:**
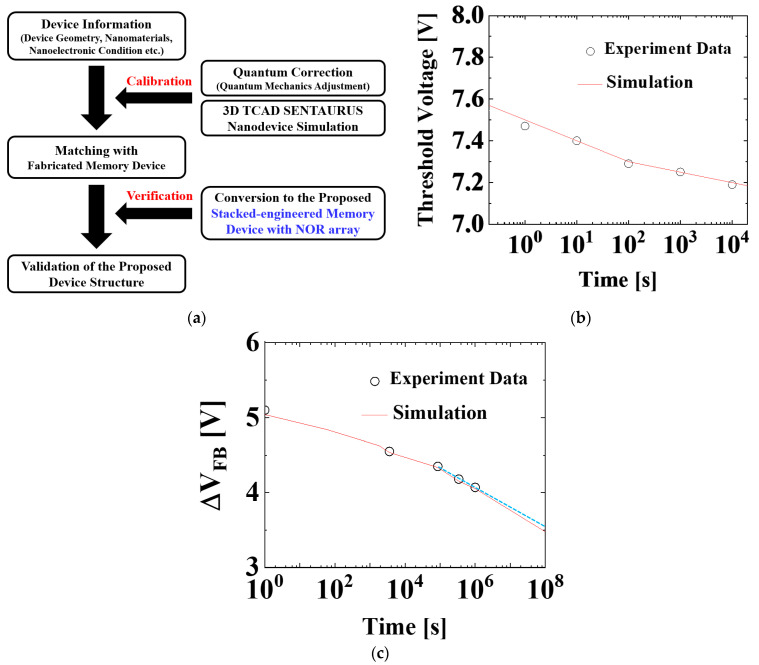
(**a**) Illustration that summarizes overall workflow of this paper; (**b**) calibration results based on the fabricated TANOS device [45]; (**c**) another calibration result based on fabricated BE-TAHOS device [46]. (Sky blue dot line indicates the linear approximation of retention characteristic in the fabricated BE-TAHOS device.).

Figure 2a illustrates the overall workflow of this paper. The calibration of memory device is performed with the fabricated memory devices [45,46], and then gate dielectric layers of SiO_2_/Al_2_O_3_/SiO_2_ is incorporated. Thereafter, validation of the proposed memory device structure is conducted in terms of retention characteristics and inhibition in the NOR flash array.

During the calibration process, quantum correlations are carefully conducted for *I*_DS_-*I*_GS_ calibration, and retention calibration is performed under Synopsys Sentaurus™ three-dimensional (3D) TCAD simulation [47]. For details, we adopted various mobility models including the PhuMob mobility model, Enormal (Lombardi) mobility model, and thin-layer mobility model to consider interfacial surface calibration roughness scattering and Coulomb scattering. Firstly, *I*_DS_-*I*_GS_ calibration is performed by carefully adopting the velocity saturation model, quantum model, and gate work function (WF). Secondly, retention characteristics are carefully calibrated with the fabricated memory devices. Figure 2b,c show our simulation results are well fit with the measured data of retention characteristics in the fabricated TaN/Al_2_O_3_/Si_3_N_4_/SiO_2_/Si (TANOS) device and BE-TAHOS device.

## 3. Results and Discussion

### 3.1. Demonstration of NOR Flash Array with the Proposed Memory Device Structure

Before demonstrating the retention enhancement from the proposed structure, the structure of the proposed memory device must be analyzed. In our proposed device structure, there are two major technological changes.

**Figure 3:**
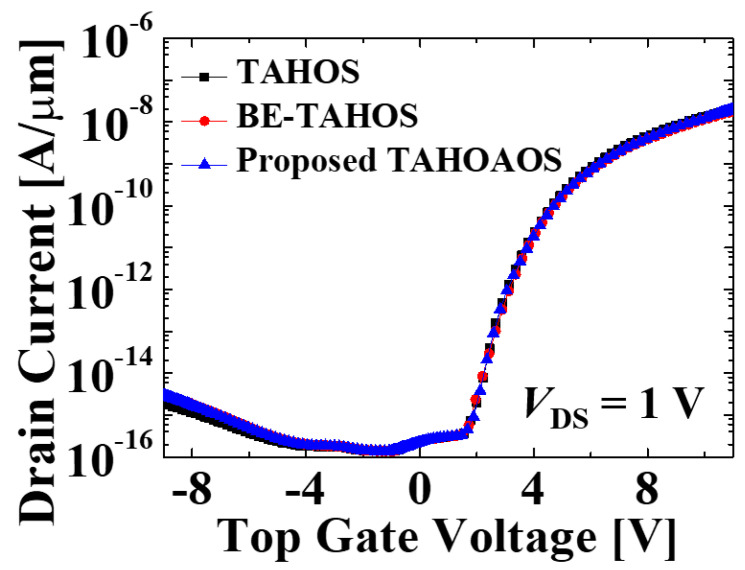
Basic transfer characteristics of three different device structures. These transfer characteristics show that our simulation is well designed with the same EOT thickness.

First, the tunneling oxide layer is technically changed for increasing physical thickness and maintaining the same EOT of 3 nm at the same time (the exact thicknesses are shown in Table 1). Since the EOT of the three structures is the same, the initial transfer characteristics are almost the same, as shown in Figure 3.

**Figure 4:**
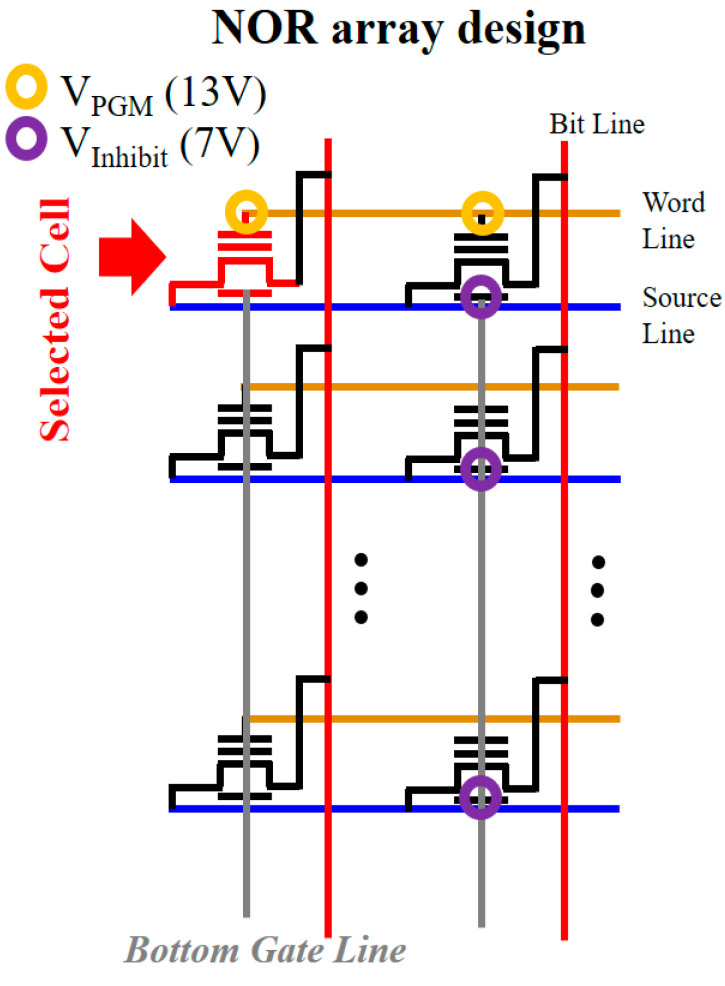
NOR array design for the proposed memory device structure. The newly added bottom gate line is perpendicular to the word line for selective programming.

Second, the bottom gate was added to suppress programming of the unselected cell and solve disturbance issues [37]. Specifically, as illustrated in Figure 4, the additional bottom gates are connected with each other by the bottom gate line, which is perpendicular to the source line and word line. From this perpendicular design between the bottom gate line and word line, it is possible to program the selected cell only and inhibit programming of unselected cells, as described in the following paragraph.

For programming, the FN tunneling mechanism is used instead of the hot-carrier injection (HCI) mechanism, which has been widely adopted for the conventional programming method in the NOR flash array [48,49,50]. This is because the conventional HCI programming consumes significant power due to a significant drain current during programming [48]. On the other hand, FN programming can lower power consumption [37] due to its lower gate current compared to the higher drain current during HCI programming [48]. Therefore, FN tunneling is adopted for programming with low power consumption.

Table 2 describes the voltage applied in the selected cell and unselected cells during programming operation under the proposed NOR array design. Programming voltage (*V*_PGM_) of 13 V and inhibition voltage of 7 V are adopted, as only 13 V can program the memory cell in high-κ–based memory devices (namely, TAHOS structure) due to low EOT of dielectric layers [18,19,20,21].

**Figure 5:**
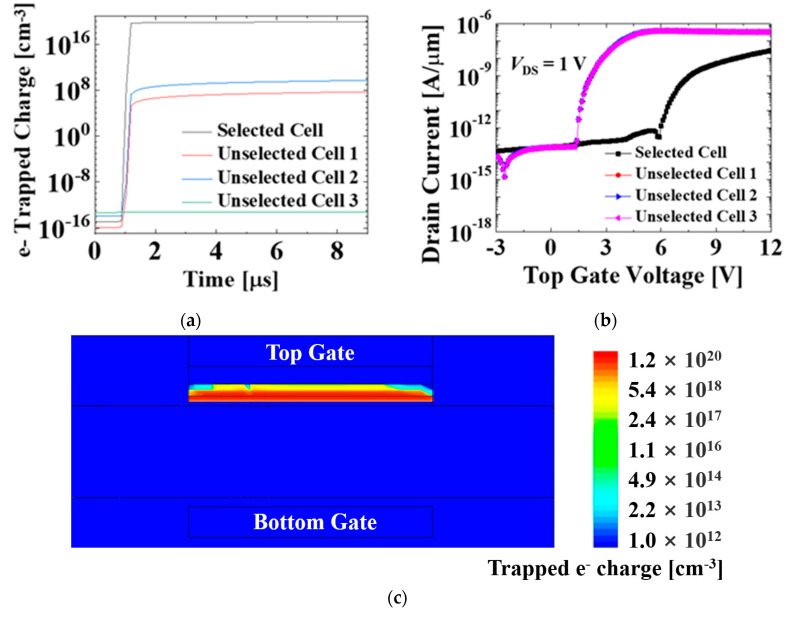
(**a**) Change of electron charge trap density during programming at the cells of the proposed NOR array design. The density of trapped electron charge becomes saturated due to limited top gate voltage. In the selected cell, the higher top gate voltage may increase the saturated density of the trapped electron charge; (**b**) transfer characteristics just after programming of the cells in the proposed NOR array design; (**c**) cross-sectional view of the selected cell with TAHOS structure that illustrates the distribution of the trapped electron charge after programming.

The different voltages are applied to the top gate and bottom gate of each cell, which serves as selective programming without disturbance issues. Consequently, as demonstrated in Figure 5, only the selected cell is programmed by FN tunneling, whereas the unselected cells are not. Regarding threshold voltage (Figure 5b), all three unselected cells show nearly zero threshold voltage shift just after programming, whereas the selected sell shows significant threshold voltage shift just after programming. This is because more than 10^16^ cm^−^^3^ trapped electron charge is needed for threshold voltage shift (Figure 5a,b) [18,19,20,21]. Therefore, it is possible to utilize our proposed structure in the NOR flash array without disturbance issues and increase the capacity of memory storage.

### 3.2. Retention Enhancement of the Proposed Memory Device Structure

**Figure 6:**
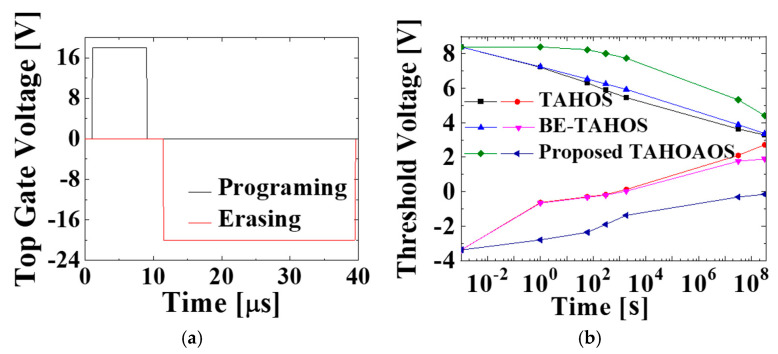
(**a**) Top gate bias during programming and erasing, and (**b**) retention characteristics of the conventional TAHOS, BE-TAHOS, and the proposed TAHOAOS structure. The high top gate voltage (17 V for programming and −21 V for erasing) is applied in order to perform fair comparison by matching initial threshold voltage at 1 micro-second. (Specifically, programming with top gate voltage of 13 V, as in Table 2, results in different initial threshold voltage [37], and hence programming with a higher top gate voltage of 17 V is performed for fair comparison.).

In order to investigate the retention enhancement of the proposed TAHOAOS structure, devices with conventional TAHOS, and BE-TAHOS, proposed TAHOAOS structures are programmed and erased with top gate voltage as described in Figure 6a. Specifically, the high top gate voltage (17 V for programming and −21 V for erasing) is applied in order to perform a fair comparison by matching initial threshold voltage (namely, threshold voltage when time is 10^−3^ s). Then, retention characteristics of each structure are analyzed for 10 years. It is shown that our proposed TAHOAOS structure maintains a significant threshold voltage window for 10 years and is very strategic for retention characteristics, as demonstrated in Figure 6b. 

**Figure 7:**
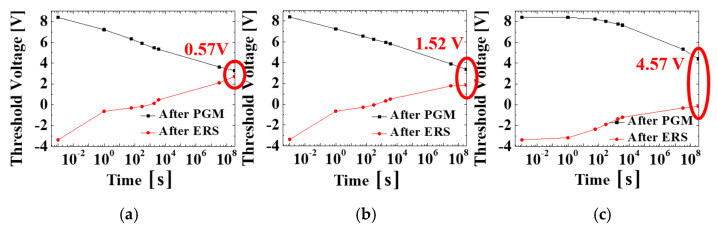
Detailed description of retention characteristics in (**a**) conventional TAHOS, (**b**) BE-TAHOS, and (**c**) proposed TAHOAOS structure.

Specifically, our proposed TAHOAOS structure maintains 4.57 V of the threshold voltage window, whereas conventional TAHOS structure only maintains 0.57 V after 10 years from programming and erasing (P/E) as illustrated in Figure 7. It is remarkable that our proposed TAHOAOS structure shows better retention characteristics (more than three times) compared to the BE-TAHOS structure.

However, there is one remarkable point in these retention characteristics. As shown in Figure 6b, the retention characteristics of conventional TAHOS and BE-TAHOS after erase operation (namely, red and pink line in Figure 6b) show barely little difference. Namely, even though retention characteristics of BE-TAHOS (pink line) is slightly better than that of conventional TAHOS (red line), the difference between them is reduced due to valence band offset.

**Figure 8:**
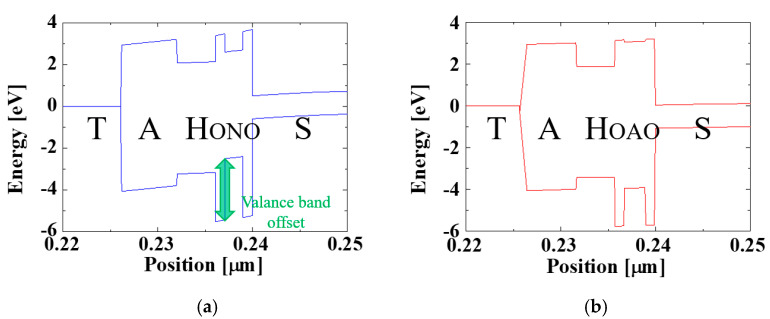
Energy band diagram of (**a**) BE-TAHOS, and (**b**) proposed TAHOAOS structure. Regarding retention characteristics, the valence band offset of BE-TAHOS (green arrow in panel a) mitigates the advantage of thicker tunneling oxide layers in BE-TAHOS. The abbreviated letters T, A, H, O, N stand for tantalum nitride (TaN, gate metal), Al_2_O_3_, HfO_2_, SiO_2_, Si_3_N_4_, respectively.

This can be explained by energy band diagram. Figure 8 shows the energy band diagram of BE-TAHOS and the proposed TAHOAOS structure with reference to previous fabricated devices of the TAHOS and TANOS structure [51]. As illustrated in Figure 8a, substantial valence band offset exists in the BE-TAHOS structure. This valence band offset helps the hole to be ejected from HfO_2_ CTL. Therefore, the advantage of thicker tunneling oxide layers in BE-TAHOS (compared to conventional TAHOS) is reduced in terms of retention characteristics.

On the other hand, the proposed TAHOAOS structure has not only thicker tunneling oxide layers but also lower valance band offset compared to BE-TAHOS (Figure 8). Therefore, regarding hole retention, the proposed TAHOAOS structure has a remarkable competitive edge, as demonstrated in Figure 6b.

**Figure 9:**
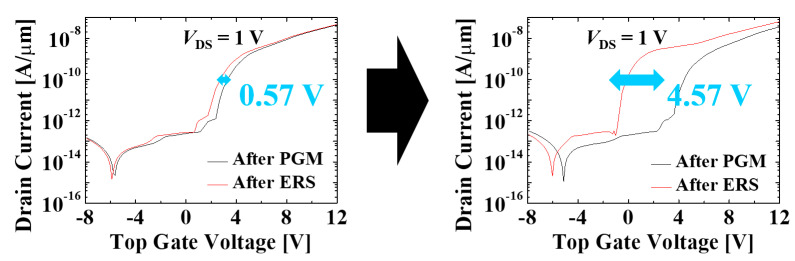
Enhancement of retention characteristics by the proposed tunneling oxide engineering. The graphs are calculated after 10 years of programming and erasing.

Figure 9 shows the transfer curves after 10 years of P/E operation in the conventional TAHOS structure and the proposed TAHOAOS structure. It is expected that our proposed structure can serve as a powerful tool for future big data markets with better reliability (retention), higher memory capacity, and low power operation (TFET-based memory [34,35,36,37,38,39,40]).

In summary, we have improved the retention characteristics with which HfO_2_-based nonvolatile charge-trapping memory has encountered [22,23,24,25], and opened up the possibility of practical application of HfO_2_-based NOR flash memory for better memory capacity.

### 3.3. Proposal for Future Research

We have proposed the design methodology for better retention characteristics and great immunity against disturbance issues by developing the TAHOAOS structure [37] on the NOR flash array. The proposed design technology is expected to improve the retention characteristics and decrease power consumption during programming (due to the programming method of FN tunneling) and during read operation (due to the TFET-based structure). Furthermore, it is expected that our newly proposed device structure with four terminals can solve the disturbance issue and make only a selected cell programmed.

However, even though our research has made considerable efforts to verify our proposed methodology, our research is basically limited to NOR flash application. We believe our proposed TAHOAOS structure can be applied beyond NOR flash application and to other fields such as 3D NAND flash and 3D AND flash. This is because our proposed technology may be applied in another domain by changing the design of the circuit. Therefore, we would like to suggest the future research topic to readers by analyzing our proposed technique in another array and another circuit design. It may be a desirable and interesting topic to develop our research with various future memory applications.

## 4. Conclusions

In this study, we propose the advanced structure for the NOR flash array with retention improvement. From the bottom gate effect, the disturbance issues are well suppressed, and it is possible to utilize the proposed structure in a NOR flash array. In addition, the threshold voltage window after 10 years of programming and erasing was considerably increased from 0.57 V to 4.57 V by incorporating Al_2_O_3_ in tunneling oxide layers. This enhancement is achieved by 1) high physical thickness of tunneling layers in the proposed structure (namely, high permittivity of Al_2_O_3_) and 2) lower valence band offset/conduction band offset in the proposed structure (namely, higher bandgap of Al_2_O_3_ compared to Si_3_N_4_). These results open up the possibility of using enriched CTL (HfO_2_) with improved retention characteristics. Therefore, the proposed TAHOAOS structure is very strategic for future highly integrated memory cells in big data markets with significant reliability enhancement.

## Figures and Tables

**Table 1:** Film thickness and channel length in conventional TAHOS, BE-TAHOS, and proposed TAHOAOS structure.

Region	Material	Thickness (nm)
Tunneling oxide	SiO_2_	3
	SiO_2_/Si_3_N_4_/SiO_2_	1/1.7/1
	SiO_2_/Al_2_O_3_/SiO_2_	1/2.3/1
Blocking oxide	Al_2_O_3_	6
Charge-trapping layer	HfO_2_	4
Bottom gate dielectric	SiO_2_	3
Channel (length)	Si	40
Channel (thickness)	Si	12

**Table 2:** The voltage applied in the selected cell and unselected cells during programming with the proposed NOR array design.

Cell Type	Top Gate Voltage (V)	Bottom Gate Voltage (V)
Selected cell	13	0
Unselected cell 1	13	7
Unselected cell 2	0	7
Unselected cell 3	0	0
